# Using computed tomography and 3D printing to construct custom prosthetics attachments and devices

**DOI:** 10.1186/s41205-017-0016-1

**Published:** 2017-08-22

**Authors:** Peter C. Liacouras, Divya Sahajwalla, Mark D. Beachler, Todd Sleeman, Vincent B. Ho, John P. Lichtenberger

**Affiliations:** 1 Department of Radiology, Walter Reed National Military Medical Center and Uniformed Services University of the Health Sciences, Bethesda, MD USA; 2 Orthotic & Prosthetic Service, Department of Rehabilitation, Walter Reed National Military Medical Center, Bethesda, MD USA; 3 Virginia Commonwealth University School of Medicine, Richmond, VA 23298 USA

**Keywords:** 3D printing, Computed tomography, Prosthetics, Additive manufacturing, Rehabilitation

## Abstract

**Background:**

The prosthetic devices the military uses to restore function and mobility to our wounded warriors are highly advanced, and in many instances not publically available. There is considerable research aimed at this population of young patients who are extremely active and desire to take part in numerous complex activities. While prosthetists design and manufacture numerous devices with standard materials and limb assemblies, patients often require individualized prosthetic design and/or modifications to enable them to participate fully in complex activities.

**Methods:**

Prosthetists and engineers perform research and implement digitally designs in collaboration to generate equipment for their patient’s rehabilitation needs. 3D printing allows for these devices to be manufactured from an array of materials ranging from plastic to titanium alloy. Many designs require form fitting to a prosthetic socket or a complex surface geometry. Specialty items can be scanned using computed tomography and digitally reconstructed to produce a virtual 3D model the engineer can use to design the necessary features of the desired prosthetic, device, or attachment. Completed devices are tested for fit and function.

**Results:**

Numerous custom prostheses and attachments have been successfully translated from the research domain to clinical reality, in particular, those that feature the use of computed tomography (CT) reconstructions. The purpose of this project is to describe the research pathways to implementation for the following clinical designs: sets of bilateral hockey skates; custom weightlifting prosthetic hands; and a wine glass holder.

**Conclusion:**

This article will demonstrate how to incorporate CT imaging and 3D printing in the design and manufacturing process of custom attachments and assistive technology devices. Even though some of these prosthesis attachments may be relatively simple in design to an engineer, they have an enormous impact on the lives of our wounded warriors.

## Background

An estimated 1.9 million amputees in the United States [1] sustained their amputations from trauma and vascular disease. The United States military care system has seen an increase in the number of amputees since the beginning of the conflict overseas. Over 1600 service members have lost limbs since 2001, with over 300 members having lost multiple limbs. Over 40,000 veterans with limb loss also receive care for their amputations within the DOD/VA system [2].

‘The art and science of prostheses’ dates back over 100 years [3], when a split tree trunk with leather straps was the best replacement leg. The methodology and technology to rehabilitate amputees has evolved over the years from muscle transplant to biomechanical manipulation and now to 3D printing technologies. Although 3D printing is a little over three decades old, it is now revolutionizing the inception and delivery of devices in rehabilitation medicine [4]. Major advances in medicine occur when medical specialties, biomedical engineers, and technologists collaborate, and the field of prosthetics is no different. Several recent manuscripts, abstracts, and case reports describing original contributions in robotic prostheses to improving 3D printing for prosthesis have been published [5–11], to. There have been many recent review articles about 3D printing and the contributions of several medical specialties ranging from physiatrists to radiologists towards improving prosthetic devices and the rehabilitation process [4, 12–15].

The United States military has been at the forefront of these new technologies and working to bring about major advances in prosthetics (e.g. extending battery life, water resistance, and improved control schemes). The majority of military amputees differ from the dysvascular amputees of the general population in that they are generally young and extremely active individuals. Military amputees are driven individuals who take part in complex activities such as kayaking, skiing, climbing, swimming, mechanical maintenance, and team sports. The age and active lifestyle of these patients are something the DOD/VA will be dealing with for the next several decades.

Currently, at WRNMMC, conventional technologies are used for socket construction. Our lab and the prosthetic service focuses on specialty attachments to reduce limitations of current prosthetic terminal device and allow individuals to once again take part in activities they desire. In this manuscript, we present examples and methods for using computed tomography and additive manufacturing to construct custom prosthetics attachments and devices.

## Methods

### 3D printing and prosthetics

3D printing, also referred to as additive manufacturing, is a process that uses a layer-by-layer approach to build an object from a digital file. In this process each subsequent layer bonds to the previous layer by means of heat, energy, or binders. This manufacturing process can be used with an array of different materials, ranging from plastics to metals. This method of manufacturing allows for production in small quantities and parts with intricate or unique geometries. Most prosthesis can be designed and manufactured with standard materials and limb assemblies that are currently available on the market. When desired specialty devices are not available, these devices can be custom designed and manufactured using digital technology and 3D printing, if an appropriate methodology has been developed.

**Fig. 1:**
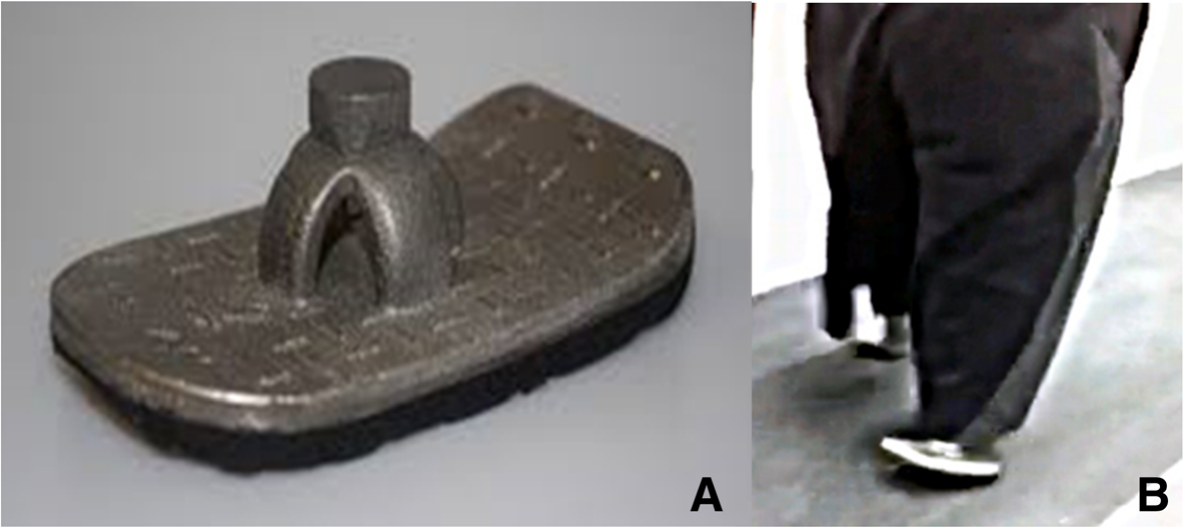
Shorty Feet. Inter-professional collaboration with prosthetics and rehabilitation medicine resulted in a custom, 3D printed, prosthetic foot. This device was designed using Computer Aided Design (CAD) and 3D printed, as 1 piece, on an electron beam melting machine, from titanium alloy. After printing, soles are bonded, to the bottom surface, to complete the device (**a**). The feet are then functional after attaching to the sockets via pyramids (**b**)

Many of these specialty prosthetics needs can be met through the combination of Computer Aided Design (CAD) in conjunction with 3D printing. For example, Shorty Feet, a device which gives bilateral amputees the ability to walk without the use of a full prosthetic, was designed entirely in CAD (Fig. 1). However, these methods are not sufficient when dealing with organic shapes and complicated shapes that require a custom solution. Previous work has been done with 3D scanners and 3D printing to create custom orthoses [13]. 3D Scanners are a great resource, however, not available at all medical institutions and highly accurate system are an added expense. Also, many 3D scanners have difficulty with clear and reflective surfaces, and can only obtain data in a direct line of sight. Due to some of these limitations of 3D scanning, computed tomography can be an additional valuable resource in creating these custom devices. The focus of this research was to develop procedures to including CT reconstructions in the design and manufacturing process of unique prosthetic componentry.

### Computed tomography utilization

Computed tomography (CT) is a powerful tool in the development of custom prosthetics. Traditional production methods for complex objects rely on labor-intensive direct measurements with calipers or rulers and the translation of that data into CAD/3D reconstruction software. Surface scanning systems using optical image capture are one option, however they typically require dedicated equipment and often require dedicated physical space. Both of these traditional methods for capturing spatial data are limited by object complexity. Specific barriers include undercuts, complex internal and external surfaces, and glossy finishes. Capturing spatial data on CT overcomes all of these barriers and has significant additional advantages. First, images are high-resolution and rapidly acquired, by-passing the need for direct measurement and eliminating translation error. Second, reconstructing scan data into digital 3D objects using specialized software allows for the seamless reconstruction of organic shapes that are not easily modeled in CAD software. Finally, acquisition techniques such as dual energy CT may also allow for the scanning of metal parts, a previous barrier to CT.

Creating devices using CT first requires acquiring a CT scan of the device’s articulating surface or area of interest. The recommended CT acquisition parameters depend on the object scanned, with z-axis resolution determined by the smallest physical detail of the object necessary to print. Generally, a reconstruction thickness of 0.625–1.25 mm will suffice. Note that these methodologies may not be sufficient for objects with critical details smaller than 0.625 mm. After acquisition of the scan, a series would then be imported into MIMICS Medical v17 (Materialise), a 3D reconstruction software that produces 3D data from the CT scan. The component is then exported and additional computer aided design would then be performed to create the device. Once the organic shape is digitized, it is possible to create custom designs that articulate with the complex geometry of the prosthetic component. Objects dependent on direct measurements would be designed in a CAD package such as Solidworks (Dassault Systemes) and then registered to the reconstruction of the articulating surface. Organic structures would be designed in a program like Freeform Plus (3D systems). The pieces and 3D reconstruction of the surface would then be registered and all the pieces aligned.

The remainder of this manuscript will discuss, in detail, three example methodologies where computed tomography was essential to the device development and construction: a weight lifting hand, hockey skate adapters, and a wine glass holder.

### Example 1: Weightlifting adapter

**Fig. 2:**
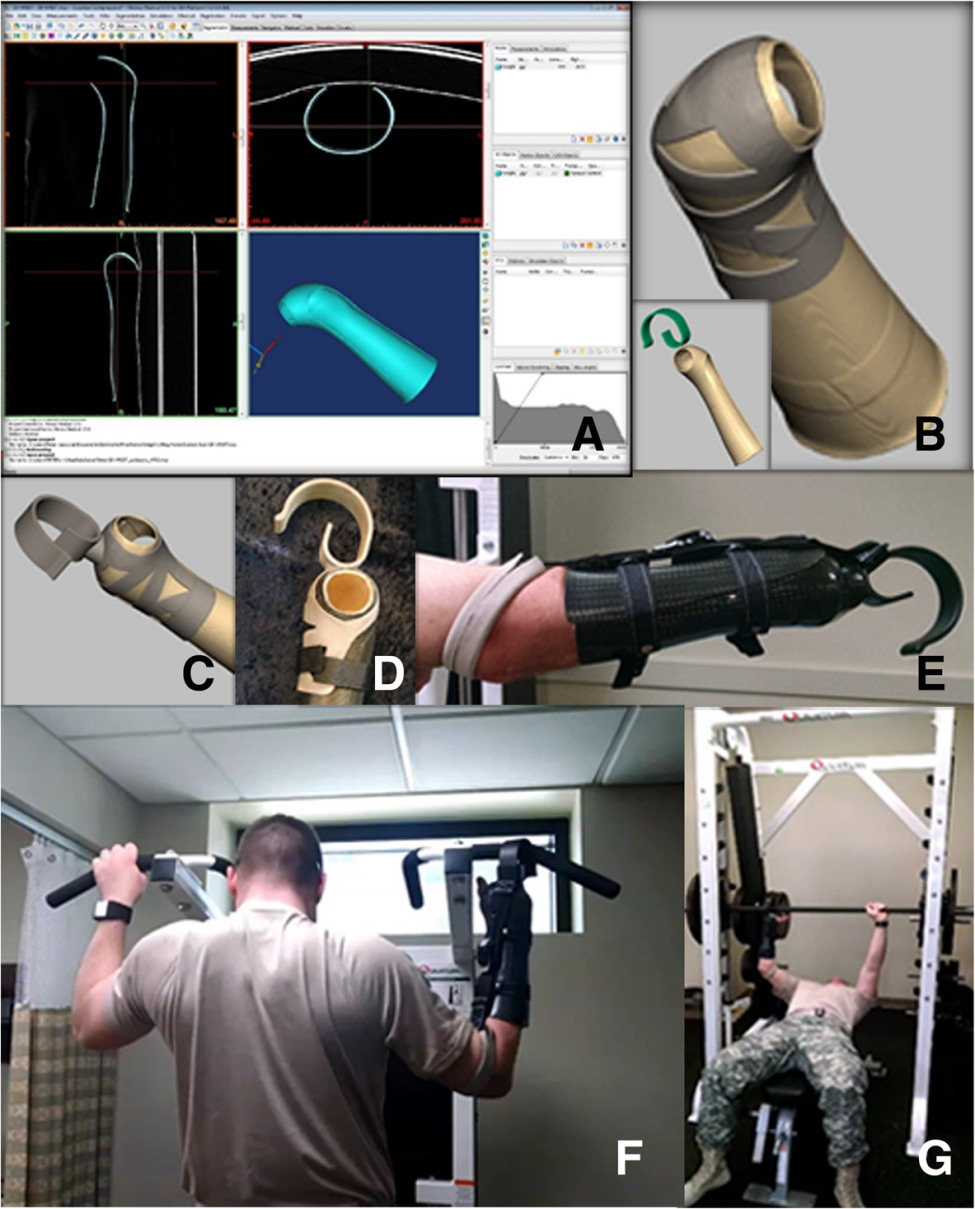
Weight Lifting Adapter. **a** Carbon fiber socket of partial hand amputee was CT scanned at 1 mm slice increments and then reconstructed. STL file was exported. **b** The CT reconstruction was used to create socket overlay. Lifting hook was designed in CAD. **c** Overlay and hook were merged into one piece. **d** Design was 3D printed in titanium alloy. 3D Print was fit tested on carbon fiber socket. **e** Caron fiber was weaved through titanium overlay and laminated into the socket. Prosthetist added a ratchet system with strap extending around the elbow for pulling exercises. **f** & **g** The attachment was successful in allowing the service member the ability perform both pushing and pulling weight lifting exercises

A partial hand amputee wanted the ability to lift weights again with both hands simultaneously and individually. The device needed to be multifunctional for both pushing and pulling exercises. The weight lifting hook device required lamination into the carbon fiber socket. The carbon fiber socket was first scanned using CT reconstructed at 1 mm slice thickness (Fig. 2a). The digital reconstruction was then exported as an STL file. After iterative consultation with the prosthetist and patient, it was determined that the weight lifting device needed to be centered on the residual limb and at the appropriate height to preserve proper biomechanics. A CAD file design was created in SolidWorks with the anatomy of a human hand in mind. The hook tries to mimic the proper barbell position for both pushing and pulling exercises. CAD files were also exported as STL files. The surface of the socket was then extruded in specific regions to allow for the carbon fiber layer to be weaved through openings and add stability (Fig. 2b). The hook was then positioned and aligned with the socket, taking height and rotation variables into consideration. The layered pattern was merged with the hook to produce a complete device customized to the patient’s prosthetic socket (Fig. 2c). The device was first 3D printed on a StereolithographyApparatus (SLA 7000), from AccuraSL 7570 (3D systems) to test fitting. Once the fit was confirmed, the device was manufactured on an Arcam Electron Beam Melting Machine A1 (ArcamAB) from titanium alloy (Fig. 2d). Carbon fiber was then used to weave and laminate the titanium hook into the prosthetic socket. The prosthetist also attached a ratchet system with a strap extending around the elbow to ensure the prosthetic socket would not migrate during pulling exercises (Fig. 2e). This custom weightlifting hand has multiple functions for a variety of pushing and pulling exercises (Fig. 2f & g).

### Example 2: Hockey skate adapters

**Fig. 3:**
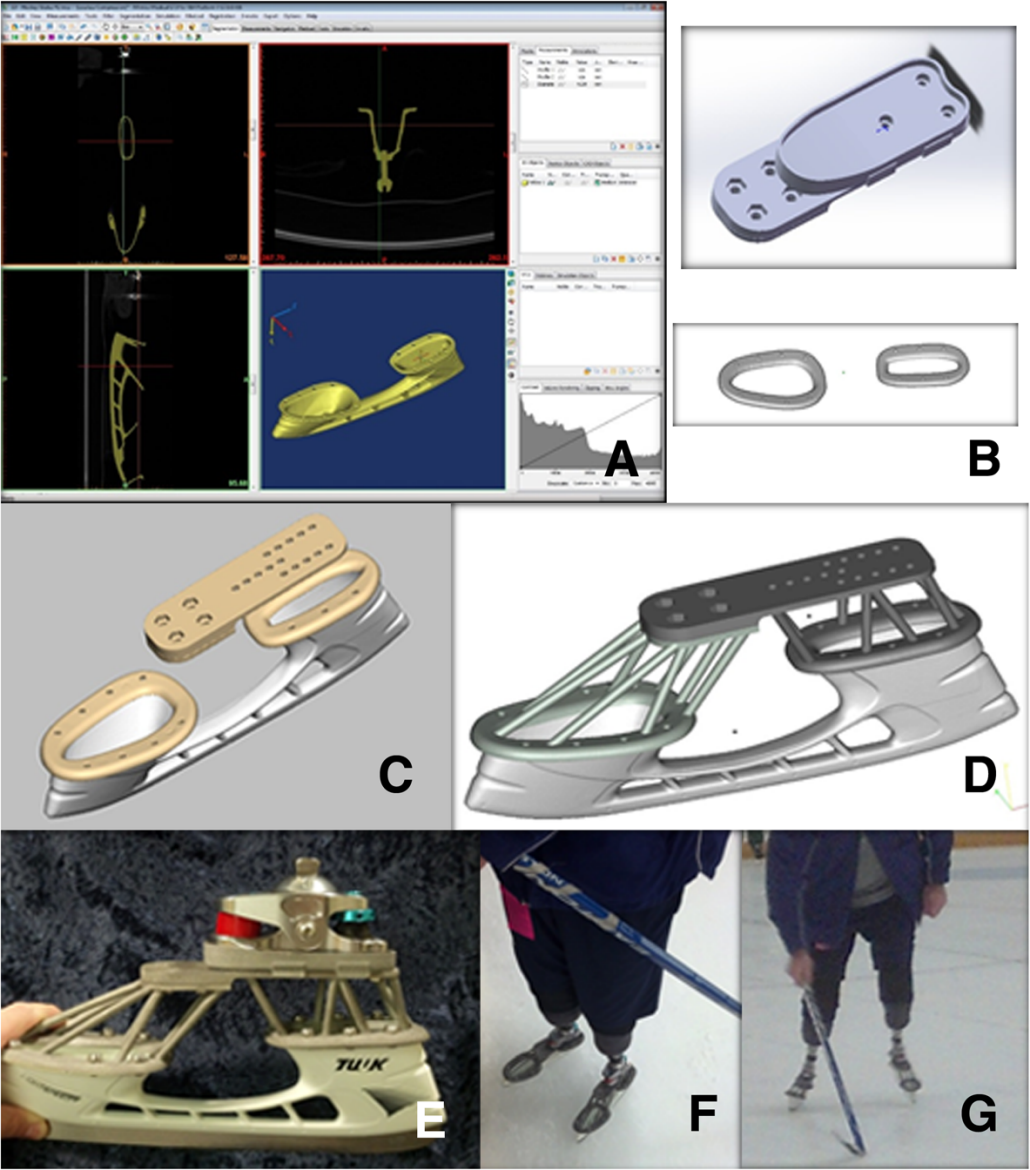
Hockey Skate Adapter. **a** Blade mount was CT scanned at 0.625 mm slice increments and then reconstructed. STL file was exported. **b** Computer Aided Design and freeform articulating surfaces created. **c** Files were aligned with the reconstruction of the skate blade mount. **d** Connecting geometry (posts) added between entities. **e** Three part assembly manufactured from titanium alloy using an electron beam melting machine. **f** & **g** Prosthetic attachments allow patients to enjoy skating or play stand up hockey

A bilateral below the knee amputee who was formerly an avid skater wanted to resume ice skating. The patient felt that they would not be able to tighten the skates enough around the prosthetic foam foot, which would reduce control and energy transfer to the skate and limited the desired sensory feedback to their residual limbs while skating. The blade mount was scanned with CT and reconstructed at 0.625 mm slice thickness (Fig. 3a). The digital reconstruction was then exported as an STL file. Note that due to the complexity and glossy finish of the surface, it would have been difficult to obtain a 3D surface scan of the blade mount. The articulating surfaces were then offset from the blade holder, and a small lip was maintained for better seating of the device. An assembly was then designed in Solidworks to hold the prosthetic ankle joint and allow for adjustability of the device (Fig. 3b). The CAD assembly was also exported as an STL file. The assembly was aligned with the articulating surface of the device and connected using numerous posts (Fig. 3c & d). The parts were merged together to create the final product. The final design was then mirrored to produce the componentry for the contralateral side. Special attention was given to the adjustability of the ankle joint and the center of gravity as the patient did not have access to the biomechanical benefit of an active ankle joint. The device was first 3D Printed on a Stereolithography Apparatus (SLA 7000), from AccuraSL 7570 (3D systems) for test fitting. Once the fit was confirmed, the device was manufactured on an ArcamElectron Beam Melting Machine (ArcamAB) from titanium alloy (Fig. 3e). The device was assembled by the prosthetist. The prosthetic attachment gave a bilateral amputee the ability to skate again (Fig. 3f & g). A special tool was also manufactured to allow the prosthetist or amputee to tighten bolts with minimal access.

### Example 3: Wine glass holder

**Fig. 4:**
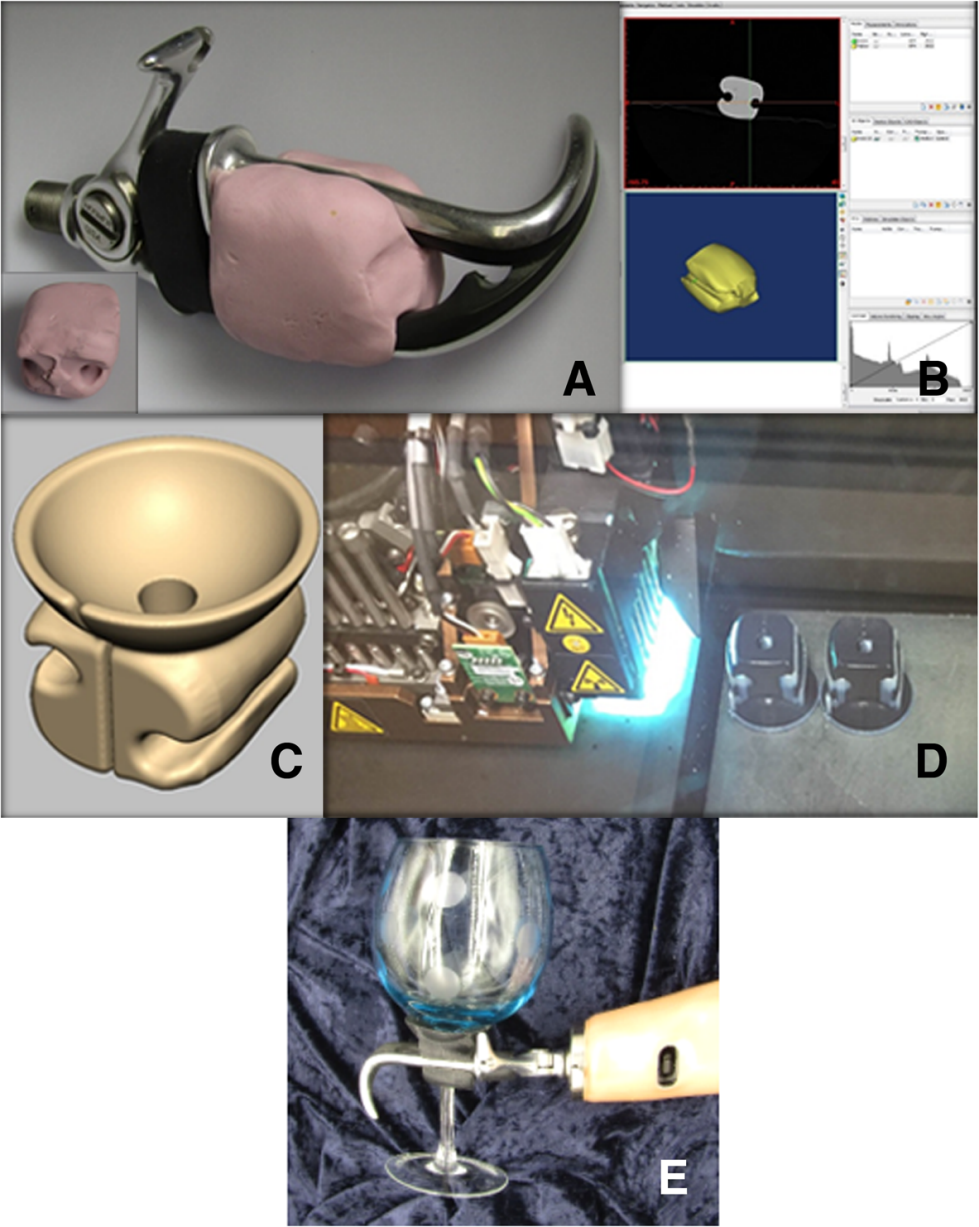
Wine Glass Holder. **a** Lab putty was used to make impression of the space between the prosthetic hook. **b** Putty impression was scanned using computed tomography (CT) at 5 mm slice increment and then reconstructed. STL was exported. **c** The cup, hole, and slit added to the design. **d** Device was 3D printed using multi-material jetting machine utilizing flexible material. **e** Prosthetic attachment enables an amputee to enjoy wine again

A 34-year old man status post upper extremity amputation had difficulty holding a standard wine glass due to the limitation of their terminal device. The prosthetic hook either applied too much pressure to the stem of the glass or the glass would wobble in the empty space between the two prongs. In order to resolve this issue, a mold of the empty space was created by placing lab putty in the open hook. The hook was then closed, allowing the putty to fill the void between the two prongs, creating an impression of both prongs (Fig. 4a). The set mold was then removed and scanned using CT at 5 mm slice increments. While 5 mm slice thickness is larger than the typical requirements, this level of detail was sufficient to serve as a basis for design. The scan was reconstructed in MIMICS Medical v17 and exported as an STL file (Fig. 4b). Note that due to the undercuts of the object, an accurate 3D surface scan would be difficult to obtain. The STL file was verified and imported into FreeFormPlus 2013 (3D Systems). A cup was added to support the bowl of the wine glass, and a 9 mm hole and slit were added for wine glass insertion (Fig. 4c). The final device was manufactured on an Objet500 Connex2 3D printer (Stratasys). The device was made from a combination of Vero White Plus and Tango Black Plus to obtain the appropriate durometer for the device (Fig. 4d). This prosthetic device enabled the amputee to enjoy wine again (Fig. 4e).

## Results and discussion

In this manuscript, we researched and presented several methodologies to construct personalized examples highlighting the benefits CT reconstructions and the 3D printing process provide in successfully manufacturing prosthetic devices in limited quantities. These are but a few examples of the applications of this technique. Three-dimensional reconstructions from computed tomography create an accurate starting geometry for designing custom prosthetic attachment and devices when barriers to traditional design processes and production methods exist. Once 3D reconstructions were obtained anywhere from thirty minutes (Wine Glass Holder) to three hours (Hockey Skate Adapters) were spent on the digital design process. 3D Printing has created new opportunities for the production of prosthetic devices and allows for the creation of unique, customized devices. Some limiting factors of these developed methodologies include the availability of equipment and materials, software, scanners, CT scan expenses, 3D printers, and experienced staff.

Materials for these prosthetic components were chosen based on the technologies and materials available at the institution. Preliminary results show that the titanium alloy used has allowed for large safety factors, greater than 2.5, on prosthetic attachments analyzed. Future endeavors include 3D printing a mold for to manufacture the Wine Glass Holder from silicone rubber. For less complex designs conventional machining could also be utilized if the facility had in-house capabilities or outsourcing could be more economical. For these particular project and other similar projects, the cost of outsourcing these components would have been higher than the cost of in-house production. CT scanning cost will differ by the institution; however, these objects do not require the radiologist reading.

## Conclusion

Using CT in conjunction with digital design and 3D printing can be utilized to create custom rehabilitation devices. Facility resources and knowledge can be limiting factors. 3D printing has created new opportunities previously unavailable to prosthetics, occupational therapy, and assistive technology departments. This methodology continues to be utilized when conventional techniques are limiting or suboptimal. Interprofessional collaboration, imaging, and digital manufacturing expertise are vital to the successful form, fit, and function of these devices.
